# Using sentiment analysis to study the relationship between subjective expression in financial reports and company performance

**DOI:** 10.3389/fpsyg.2022.949881

**Published:** 2022-07-22

**Authors:** Ni Zhong, JunBao Ren

**Affiliations:** ^1^Financial Management Division, Shandong Tudi Development Group Co., Ltd., Jinan, China; ^2^China Construction Bank Shandong Branch, Jinan, China

**Keywords:** financial reporting, subjective expression, company performance, sentiment analysis, multi-dimensional relationship

## Abstract

In recent years, with the development and progress of text information research, the disclosure of non-financial and qualitative information has often be found to have an incremental function. Financial reports, including financial statements and other relevant information, provide important insights on an enterprise’s financial status, operating results, and cash flow. Faced with a large number of financial reports, readers often do not know where to start, and as financial statements are prepared based on past transactions, they cannot fully reflect the past, present, and future economic conditions of the company. Information asymmetry and uncertainty make the text mining of financial reports of great significance to enterprise stakeholders. Accordingly, this paper takes financial reports as the research object and builds a research framework on the relationship between subjective expression in financial reports and company performance. Through natural language processing, sentiment analysis, and other text-mining technologies, the paper quantifies the subjective expression in financial reports and introduces intermediaries. Variables, moderating variables, and control variables are used to construct a multiple regression model. The empirical results show that the underlying emotional tendencies in subjective expressions substantially impact on the future development of listed companies. This paper enriches understanding of the multi-dimensional relationship between financial report text and company performance, and provides ideas for further exploring this relationship. It is of great practical significance to help them make rational decisions and ensure the normal operation of the company and the preservation and appreciation of capital.

## Introduction

The financial report is like a comprehensive business card, providing accounting information on the issuing company that reflects its financial status, operating results, and cash flow. Primarily, the financial statements objectively and fairly reflect the company’s economic activities and transactions. By contrast, other parts of the financial report, such as off-balance sheet notes, are prepared using a series of estimates, judgments, and models that capture uncertainties. Providing performance information that may be short- or long-term, financial or non-financial, internal or external, and from different stakeholder perspectives, these parts of the report help users make economic decisions. Among prior studies of financial reports and corporate performance, Henry’s (2006) empirical research revealed that the emotion in earnings news releases had a certain impact on investor behavior (Henry, 2006). Tetlock (2008) found that descriptions with negative affective words are related to negative company performance (Tetlock et al., 2008), while Li (2010) reported that partial sentiment in management discussion and analysis (MD&A) can mitigate the mispricing of accruals. This means that when managers price accruals with a “warning” emotion, their pricing tends to be fair (Li, 2010). Bollen and Huina (2011); Loughran and McDonald (2011), Samuel et al. (2011), and Chang et al. (2012) and others found that positive text sentiment is significantly positively correlated with the company’s future performance. McKay Price et al. (2012) proposed that the language tone of conference calls is an important predictor of abnormal returns and transaction volume (McKay Price et al., 2012). This shows the need to continue in-depth research on text information. Hajek et al. (2013) combined sentiment indicators from financial reports with financial data indicators to improve the accuracy of stock price prediction models (Hajek et al., 2013). Meanwhile, Kearney and Liu (2014) extracted text information from various sources, studied the differences between content and methods, and found the impact of text sentiment on the individual, corporate behavior, and market levels (Kearney and Liu, 2014).

Emotional characteristics are a unique value of text information. The author’s opinions and attitudes and other emotional information can be expressed through the words, grammar, and rhetoric of the text. This information is hidden in the text and the author may not even be consciously aware of it, meaning such information has special value (Qiujun and Chaoqun, 2011). Although financial reports are somewhat formulaic and standardized, there is still information in how meaning is expressed in the text. Loughran and McDonald (2011) developed a financial sentiment dictionary (hereafter, “L&M dictionary”), which is suitable for English-language research and can reduce the misclassification caused by the domain problem of text in sentiment analysis (Loughran and Mcdonald, 2011). In a study of British companies’ annual reports, Yekini et al. (2015) used the frequency of positive words as defined in the L&M dictionary to measure the emotional tone and verified the market’s reaction to the positivity of financial report narratives (Yekini, 2016). Similarly Hajek (2017) used the L&M dictionary to conduct a collocation analysis of positive words combined with negative words in the annual reports of American companies, and measured the ratio of various emotional words to the total number of emotional words. They found that after incorporating sentiment features, the abnormal stock returns could be much more accurately predicted (Hajek, 2017). In a study of the sentiment trend of Chinese companies’ annual reports, Song et al. (2018) annotated sentences of reports in different industries and used the support vector machine method for sentiment classification (Song et al., 2018). Bakarich et al. (2019) studied the relationship between the tone of annual reports and the life cycle of enterprises; they found that the tone of annual reports was more positive, while the tone of enterprises in recession was the opposite (Bai et al., 2019; Beretta et al., 2019; Bian et al., 2019; De Souza et al., 2019; Campbell et al., 2020).

As technology continues to advance, scholars are experimenting with different techniques and models for analyzing the relationship between financial reporting and corporate performance. Due to information asymmetry, capital markets pose the risk of adverse selection and moral hazard. Ordinary shareholders and even corporate shareholders often know little about a company’s real business status. When existing or potential investors read a large number of corporate financial reports, it is often difficult to discover the underlying performance information. Falsification of financial reports is the best illustration. No matter how brilliant the financial data might appear, the underlying performance information reflects the company’s real financial situation. For this reason, applying sentiment analysis technology to financial reports and establishing a set of methods to effectively identify a company’s real performance has important practical significance for both management and investors.

## Sentiment analysis methods

### Natural language processing

**FIGURE 1:**
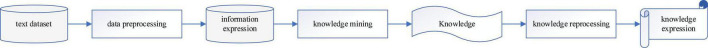
Basic text mining model.

After obtaining text information from a company’s financial report, it is necessary to further process and quantify the text using text-mining technology. As an extension of data mining, text mining is the extraction of implicit and imperceptible information with potential commercial value from semi-structured or even unstructured mass text information. Text mining is an extension of data mining, that is, data mining from the text information. After first segmenting the unstructured text, the text feature information is then extracted and stored in a structure similar to a relational database. Next comes the process of learning and knowledge pattern extraction, whereby data mining techniques (e.g., classification, clustering) are used to obtain valuable and important information. A basic text mining model is depicted in Figure 1.

The text of financial reports is processed using stuttering word segmentation, an excellent component based on Python. There are currently three word-segmentation modes, which can be adapted to different needs. The main algorithm implementation principle of stuttering word segmentation is based on the word search tree structure. It is a kind of hash tree that can realize efficient vocabulary scanning, find possible words in all text in the sentence, use these words to form a directed acyclic graph, then find the path with the largest word probability in the sentence and calculate the optimal segmentation combination according to word frequency. For unregistered words, the hidden Markov model is used for identification and the Viterbi algorithm is used. Stuttering word segmentation mainly adopts dictionary-based technology. There are three supported word-segmentation modes: (a) The precise mode, which divides the sentence into the most accurate word-segmentation process, which is suitable for text analysis; (b) the full mode, in which all possible words in the sentence are segmented and the segmentation rate is very high, but words cannot be accurately segmented according to the context and the ambiguity problem cannot be overcome; and (c) the search engine mode, which builds on the precise mode by segmenting some words again to improve the word-segmentation recall rate. Importantly, stuttering word segmentation supports traditional Chinese text segmentation and also supports custom dictionaries as needed. The stutter participle currently has Python, JAVA, C++, and Node.js versions. This paper uses the programming language R to segment financial reports. After first installing the stuttering word segmentation developer toolkit in in the R operating environment, we next performed word-segmentation processing, then cleaned the word segmentation results, and finally generated word frequency statistics.

### Measure of subjective expression

Using natural language processing technology, we extracted subjective sentences from financial reports and marked the overall emotional tendency of each sentence. Because a sentence may describe more than a single aspect of the situation and express more than one emotion, the object of subjective expression is extracted by extracting and description. On average, each subjective sentence contains two sets of “object-description” subjective expressions. The expression of subjective emotions is complex, often affirming one aspect and criticizing others at the same time, but the emotional tendency of the whole sentence can be discerned. As the emotional expression in part of a sentence may not be consistent with the emotional tendency of the whole sentence, we marked emotional tendencies of not only whole sentences but also each “object-description” set, as shown in Table 1.

**TABLE 1:** Examples of subjective expression recognition in financial reports.

Sentence emotion	Sentence	Object	Describe	Describe the emotion
Negative	Mainly attributable to the significant year-on-year decline in the prices of chemical products and the year-on-year decline in the performance of the chemical sector.	Product price	Decline	Negative
Negative	These may have a greater impact on the company’s production, operation, and benefits.	Production and operating and efficiency	Greater impact	Negative
Negative	Affected by changes in the market situation, the company’s active adjustment of product structure, and the voluntary shutdown of some old models, the company’s production and sales scale declined during the reporting period.	Production and sales scale	Slip	Negative
Positive	The company’s stable management style, prudent financial management, and good credit accumulation have been recognized by international investors.	Business style	Steady	Positive
		Financial management	Careful	Positive
		Credit accumulation	Good	Positive
Positive	Facing the complex external environment and increasingly fierce industry competition, the company insists on “quality growth,” with steady growth in scale and continuous improvement in operating efficiency.	External environment	Complex	Negative
		Industry competition	Increasingly intense	Negative
		Scale	Steady growth	Positive
		Operational efficiency	Constantly improving	Positive

**FIGURE 2:**
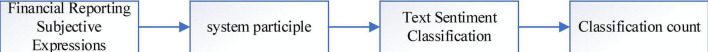
Text data sorting process.

Focused on subjective expression in financial reports, this paper uses a dictionary-based method for evaluating sentiment words. The classification of sentiment words is mainly determined by the selected dictionary. At present, there are mainly two suitable dictionaries for use with Chinese text: the National Taiwan University Semantic Dictionary (hereafter “NTU dictionary”) and the HowNet Sentiment Dictionary (hereafter “HowNet dictionary”). The NTU dictionary comprises 2,812 praise words and 8,276 derogatory words, whereas the HowNet dictionary includes two categories of praise and criticism, including 9,193 text evaluation words and 9,142 English evaluation words. It provides not only positive and negative sentiment words but also positive and negative evaluation words and degree adverbs. We therefore selected the HowNet dictionary to sort, classify, and count the sentiment words extracted from financial reports. The text data sorting process is shown in Figure 2.

After completing the download statistics of the data analysis samples of financial reports, we classified and sorted the word segmentation according to the HowNet dictionary. As the negative value phenomenon in the rooted part (where there are more negative emotional words than positive emotional words) causes missing or wrong values in the measurement formula, we avoid this by adding the absolute value and improving the measurement, as shown in formula (1):


(1) $$J\,Q\,G=\sqrt{\left|\frac{Z\,M\,Q\,G-F\,M\,Q\,G}{Z\,M\,Q\,G+F\,M\,Q\,G}\right|}$$

where *JQG* measures the net emotion; *ZMQG* measures the proportion of all sentiment words that are positive in the text of the *t*-th financial report; and *FMQG* measures the proportion of all sentiment words that are negative in the text of the *t*-th financial report. If the positive emotion proportion is greater than the negative emotion proportion, the value of *JQG* will be larger, indicating that the net emotion of the text tends to be optimistic; conversely, if the negative emotion proportion is greater than the positive emotion proportion, the value of *JQG* will be smaller, indicating that the net emotion of the description text tends to be pessimistic.

### Regression analysis

**FIGURE 3:**
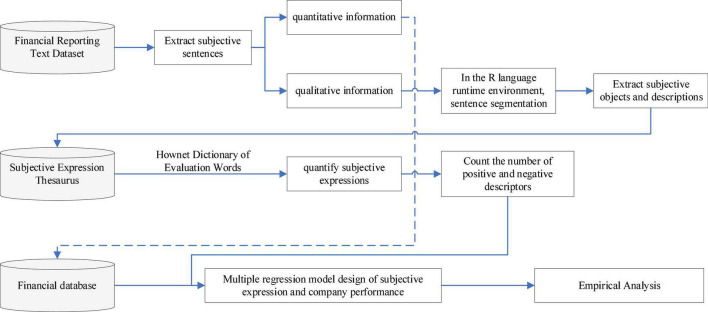
Research framework for exploring the relationship between subjective expression in financial reports and company performance.

A purely statistical test of subjective expression in financial reports can only reveal that events have a certain impact on corporate performance, and cannot accurately measure the magnitude of the impact. For the latter purpose, it is necessary to carry out a regression analysis. This paper establishes a regression model to investigate the relationship between the emotions expressed in a company’s financial report and that company’s future performance (measured by return on equity). The specific research framework is shown in Figure 3.

## Sentiment analysis model design

### Hypotheses development

Subjective expression in financial reports may influence the company’s future performance via its reference value for investors. For most investors, confidence in a company will be boosted by text information in financial reports conveying positive emotions and predicting much room for improvement in future performance. In turn, investors’ greater confidence affects their decision-making, leading to a rise in the company’s stock price and a positive impact on the company’s future performance. By contrast, the expression of negative emotion regarding a company’s financial status likely leads investors to doubt the company’s ability to develop in the future; such weakened confidence in the company’s prospects likely increases investors’ inclination to withdraw their investment from the company, thereby negatively impacting on the company’s future performance. Based on these considerations, this paper proposes the following research hypothesis:

H1: The net sentiment expressed subjectively in financial reports affects investor confidence and, in turn, the company’s future performance, so investor confidence plays an mediating role.

Earnings per share is generally an important indicator to measure company profitability. It shows that earnings per share is positively correlated with company performance, and people are more sensitive to the negative tone. Although written sentiment in financial reports is relatively neutral, negative sentiments are expressed implicitly and have been shown to be negatively correlated with the company’s future performance. Therefore, it is preliminarily inferred that earnings per share weakens the negative correlation between the net sentiment of the text and the company’s future performance. The following research hypothesis is thus proposed:

H2: Earnings per share plays a moderating role between the net sentiment of the text and the company’s future performance by weakening their negative correlation.

### Variables

We downloaded from company websites the financial reports of representative companies with a medium-to-high market value, such as Oriental Fortune and Snowball. After selecting a sample of over 920 companies, we used the word-segmentation system to segment the sentences. Then, according to the HowNet dictionary, we extracted positive and negative sentiment words. Table 2 describes the classification method of the HowNet dictionary. This paper will classify sentiment words in financial report texts according to this classification method. All data to analyze financial performance were downloaded from the databases of Guotai Junan and Ruisi.

**TABLE 2:** Summary of subjective expression in financial reports.

Emotion of words	Total number	Word description
Positive	854	Good, steadily improving, improving
Negative	1,423	Downward, slight, down

The variables used in this paper are defined in Table 3.

**TABLE 3:** Variable definitions.

Type	Description	Name	Definition and calculation formula
Dependent	Company’s future performance	*ROE_*iT*+1_*	Return on equity
Explanatory	Text positive emotion	*ZMQG*	Number of positive emotional words in the total number of emotional words
	Text net emotion	*JQG*	$J\,Q\,G=\sqrt{\left|\frac{Z\,M\,Q\,G-F\,M\,Q\,G}{Z\,M\,Q\,G+F\,M\,Q\,G}\right|}$
Mediator	Investor confidence	*IC*	*IC* = 0.7604 * *Growth* + 0.7182 * *YrPB* + 0.6163 * *INST* where *Growth* represents the growth rate of main business revenue, *YrPB* represents the price-to-book ratio, and *INST* represents the proportion of institutional investors
Moderator	Earnings per share	*EPS*	
Control	Scale	*SIZE*_*T*_	Natural logarithm of total assets
	Listing period	*AGE*	
	Growth	*MB*	Net profit growth rate
	Market return	*YRET*	Return on invested capital

### Regression model design

Referring to the models of Xie and Lin (Deren and Le, 2014, 2015, 2016), this paper establishes four models: Models 1, 2, and 3 mainly test H1, while Model 4 mainly tests H2.

Models 1, 2, and 3 are proposed as formulas (2)–(4):


(2) $$R\,O\,E_{i\,T+1}=\mu_{0}+\mu_{1}\,J\,Q\,G+\mu_{2}\,M\,B+\mu_{3}\,Y\,R\,E\,T+\mu_{4}\,L\,N\,S\,I\,Z\,E+\mu_{5}\,A\,G\,E+\xi_{i\,T}$$

(3) $$R\,O\,E_{i\,T+1}=\alpha_{0}+\alpha_{1}\,I\,C+\alpha_{2}\,M\,B+\alpha_{3}\,Y\,R\,E\,T+\alpha_{4}\,L\,N\,S\,I\,Z\,E+\alpha_{5}\,A\,G\,E+\xi_{i\,T}$$

(4) $$R\,O\,E_{i\,T+1}=\beta_{0}+\beta_{1}\,J\,Q\,G+\beta_{2}\,I\,C+\beta_{3}\,M\,B+\beta_{4}\,Y\,R\,E\,T+\beta_{5}\,L\,N\,S\,I\,Z\,E+\beta_{6}\,A\,G\,E+\xi_{i\,T}$$

Model 1 includes *JQG* as the explanatory variable, rather than *ZMQG*. It should be noted that since this paper does not propose any hypotheses on how the negative sentiment of text may influence the company’s future performance, it is mainly because the text of the financial report is after sorting out the text information, the negative emotional words in it will inevitably be weakened. In addition, emotional expression in the text is more subtle, which will inevitably affect research on the direct correlation between negative emotions and the company’s future performance. However, as people are highly sensitive to negative emotions, this paper takes account of their impact when studying the relationship between net sentiment and company performance.

For H1 to be supported empirically, the verification Model 1 *JQG* is negative and significant, the verification *IC* is positive and significant, and the verification Model 3 after adding *IC*, *JQG* is not obvious, and *IC* is significant.

To test H2’s prediction on the moderating role of earnings per share between the net sentiment of the text and the company’s future performance, we include the interaction term of *JQG* and earnings per share (*EPS*) in Model 4:


(5) $$R\,O\,E_{i\,T+1}=\theta_{0}+\theta_{1}\,J\,Q\,G+\theta_{2}\,J\,Q\,G\times E\,P\,S+\theta_{3}\,M\,B+\theta_{4}\,Y\,R\,E\,T+\theta_{5}\,L\,N\,S\,I\,Z\,E+\theta_{6}\,A\,G\,E+\xi_{i\,T}$$

### Sample selection and data sources

#### Sample selection

The text information data are taken from the sample companies’ 2019 financial reports. The two explanatory variables, positive sentiment (*ZMQG*) and net sentiment (*JQG*), are both measured using these data. As we are interested in how sentiment affects future performance, the dependent variable is measured using the return on equity in 2020 (*ROE*_*iT* + 1_). Many companies pay more attention to earnings per share as an indicator of profitability or performance, but if the growth rate of shareholders’ earnings is higher than that of after-tax profit, then net profit (return on assets) of falling. ROE measures the efficiency of investment output, and so is considered suitable to measure company performance in this study.

However, earnings per share undeniably reflects the company’s profitability to some extent. We therefore include it as a moderating variable in H2 and empirically test whether it can correlate with the net sentiment between words and the company’s future performance. Adjust between them.

The intermediary variable investor confidence (*IC*) is calculated using the formula in Table 3. Since a company’s annual report is a relatively authoritative information source, the sentiment of its text is particularly important for investors who lack information. Consequently, this sentiment affects investors’ future expectations of the company, in turn influencing their investment decisions and, ultimately, the company’s future performance. On this basis, investor confidence is included as an intermediary variable.

To account for factors likely to influence company performance, the following control variables were selected: the logarithm of the company’s total assets in 2019 (*LNSIZE*); the growth rate of the company (*MB*), measured by the growth rate of the company’s net profit in 2019; listing age (*AGE*) in 2019, measured from the year when the company went public; and market return (*YRET*), measured by the company’s return on invested capital in 2019.

#### Data sources

The text information mainly comes from the financial reports of companies such as Oriental Fortune.com and Xueqiu.com. After screening, the text information of over 920 companies was included in the analaysis. Subjective expressions were quantified, and collating counts were performed. It should be noted that owing to continuous data screening in the process of measuring different models, the number of observations is not the same for each variable; nonetheless, text data for at least 800 enterprises were empirically analyzed. Data for the dependent, mediator, moderator, and control variables were sourced from the databases of Guotai Junan and Ruisi. We mainly used EViews 7.2 for regression analysis.

## Empirical analysis

### Descriptive statistics

Table 4 shows the descriptive statistics of all variables, including the mean, median, maximum, minimum, standard deviation, and number of observations.

**TABLE 4:** Descriptive statistics of variables.

Variable	Mean	Median	Maximum	Minimum	*SD*	Number of observations
*ROE*_*iT* + 1_	0.3372	−0.0159	11.5162	−23.1938	4.3706	908
*ZMQG*	0.7524	0.7857	0.9500	0.0000	0.1285	908
*JQG*	0.9672	0.7559	0.9486	0.0000	0.1981	891
*IC*	16.1719	11.0086	62.6143	−6.9037	3.8377	877
*EPS*	0.2664	0.20000	4.15	−4.4800	0.5234	908
*LNSIZE*	21.7363	21.6154	28.4133	14.9416	0.5862	908
*AGE*	9.8369	7.0000	25.0000	0.0000	6.7883	926
*MB*	−17.7670	12.4678	45.4048	−10.0587	3.5227	908
*YRET*	6.5855	6.1556	64.4569	−16.0109	1.0638	908

The mean of the dependent variable *ROE*_*iT* + 1_ is 0.3372 but its median is −0.0159, indicating that it has both positive and negative values. Among the explanatory variables, the mean of *ZMQG* is 0.7524, the median is 0.7857, and the maximum is 0.9500. These values show that positive emotion accounts for a large proportion of text information, and the mean and median values are relatively average. *JQG* has a mean of 0.9672, a median of 0.7559, a maximum of 0.9486, and a minimum of 0.0000. The mediator variable *IC* has a mean of 16.1719 and a median of 11.0086. Meanwhile, the moderator variable *EPS* has a mean of 0.2664, a maximum of 4.1500, and a minimum of −4.4800, indicating that earnings per share varies greatly among the sample companies. For the control variables, the mean of *LNSIZE* is 21.7363, its median is 21.6154, its maximum is 28.4133, and its minimum is 14.9416; *AGE* has a mean of 9.8369, a median of 7.0000, and a maximum of 25.0000; the mean of *MB* is −17.7670 but its median is 12.4678, indicating that a large number of companies have a poor net profit growth rate; *YRET* has a mean of 6.5855, a median of 6.1556, a maximum of 64.4569, and a minimum of −16.0109.

### Correlation analysis

We tested the correlations between all variables. The results are shown in Table 5.

**TABLE 5:** Correlation analysis results.

	*ROE*	*ZMQG*	*JQG*	*IC*	*EPS*	*MB*	*AGE*	*LNSIZE*	*TRET*
*ROE*	1								
*ZMQG*	−0.082*	1							
	0.014								
*JQG*	−0.015*	0.639**	1						
	0.656	0.000							
*IC*	−0.049	0.037	0.030	1					
	0.144	0.273	0.375						
*EPS*	0.001*	−0.013	−0.022	0.077*	1				
	0.770	0.685	0.508	0.022					
*MB*	−0.001	−0.016	−0.017	0.052	0.126**	1			
	0.782	0.637	0.602	0.123	0.000				
*AGE*	0.046	0.031	0.035	−0.104**	−0.168**	−0.048	1		
	0.165	0.351	0.289	0.002	0.000	0.150			
*LNSIZE*	−0.052	−0.0089**	0.062	−0.152**	0.100**	−0.032	0.386**	1	
	0.118	0.007	0.063	0.000	0.003	0.331	0.000		
*TRET*	−0.052	−0.031	−0.056	−0.050	0.590**	0.092**	−0.122**	0.077	1
	0.116	0.350	0.094	0.136	0.000	0.006	0.000	0.021	

*Significantly correlated at the 0.05 level (two-sided).

**Significantly correlated at the 0.01 level (two-sided).

As shown in Table 5, the dependent variable *ROE*_*iT* + 1_, the explanatory variables *ZMQG* and *JQG*, the mediator variable *IC*, and the moderator variable *EPS* are basically unrelated. Therefore, the regression model in this paper is not greatly affected by multicollinearity.

### Regression results

Analysis of the Subjective Expression of Financial Reports and the Results of Multiple Regression of Corporate Performance.

#### Mediating effect of investor confidence

This group of experiments uses CASIA data to compare the four models. To prove the ability of the attention mechanism to identify time series features, the CNN-LSTM model proposed in the literature Peng et al., 2022 is used for the first set of comparisons. The second set of comparisons uses the AC-BiLSTM model Dong et al., 2020, and the third set of comparisons uses the Self-Attention-BiGRU model Qiu et al., 2020 to ensure accuracy and stability.

Table 2 shows the comparison of sentiment classification parameters of the CNN-LSTM, AC-BiLSTM, Self-Attention-BiGRU, and DAtt-CBLSTM models with the CASIA data.

In the empirical results reported in Table 6, the coefficient on *JQG* in Model 1 is −17.6787, significant at the 5% level; the coefficient on *IC* in Model 2 is −0.0662 but not significant; in Model 3, the coefficient on *JQG* is −18.3518, significant at the 5% level, and the coefficient on *IC* is −0.0661 but not significant.

**TABLE 6:** Regression results of the mediating effect of investor confidence.

	Dependent variable: *ROE*_*iT* + 1_		
Variable	Model 1	Model 2	Model 3
*ZMQG*			
*JQG*	−17.6787**		−18.3518**
	(−2.3574)		(−2.3826)
*IC*		−0.0662	−0.0661
		(−1.673)	(−1.6544)
*MB*	0.6561	0.8171	0.0001
	(0.1575)	(0.1945)	(0.2411)
*YRET*	−0.1588	−0.1616	−0.1287
	(−1.1308)	(−1.1312)	(−0.8880)
*LNSIZE*	−2.6936*	−3.4032**	−3.1846**
	(−1.8930)	(−2.3009)	(−2.1102)
*AGE*	0.4597*	0.4687*	0.4757*
	(1.9350)	(1.9319)	(1.9335)
N	891	877	860
F	2.7563	2.2706	2.803

*Indicates a significant correlation at the 0.05 level (two-sided); **indicates a significant correlation at the 0.01 level (two-sided).

Research hypothesis H1: Untested, indicating that the mediating effect of investor confidence between text net sentiment and the company’s future performance is not significant. Investor confidence can affect a company’s future performance in part through its impact on the company’s stock price. Deren and Le (2014) found that investors were obedient and have a significant positive response to positive emotion based on the annual performance briefing. Significantly negative reactions to negative emotions. However, they did not study whether investor reactions are related to the company’s future performance. Bochkay (2019) found that in a calm and stable stock market, investor sentiment has little effect on stock price; however, in a period of stock market turbulence, investor sentiment is difficult to rationally control because of uncertainty about future prospects, so investment behavior at this time is mainly affected by investor sentiment, which has a greater impact on the stock price.

We find no significant correlation between investor confidence and the company’s future performance. This may be because our models do not take into account the impact of the stock market environment and related financial management, such as earnings management, which are both known to affect company’s performance.

#### Moderating effect of earnings per share

Model 4 tests H2, namely whether earnings per share plays a moderating role between the net sentiment of the text and the company’s future performance.

As can be seen from the empirical results in Table 7, The coefficient on *JQG* is −19.2506, significant at the 5% level, while the coefficient on the interaction between *JQG* and *EPS* is 9.5262, also significant at the 5% level. These findings show that EPS somewhat weakens the negative relationship between *JQG* and *ROE*_*iT* + 1_, supporting H2. As shown in Table 8, earnings per share is positively correlated with company performance with a coefficient of 5.6935, significant at the 1% level.

**TABLE 7:** Regression results of the moderating effect of earnings per share.

	Dependent variable: *ROE*_*iT* + 1_
Variable	Model 4
*ZMQG*	
*JQG*	−19.2506**
	(−2.5585)
*IC*	
JQG*EPS	9.5262**
	(2.06100)
*MB*	0.4110
	(0.0099)
*YRET*	−0.2858*
	(−1.7768)
*LNSIZE*	−3.1515
	(−2.1922)**
*AGE*	0.5327
	(2.2216)
N	891
F	3.0133

*Indicates a significant correlation at the 0.05 level (two-sided); **indicates a significant correlation at the 0.01 level (two-sided).

**TABLE 8:** Relationship between earnings per share and company performance.

.	Dependent variable: *ROE*_*iT* + 1_
Variable	Model
*EPS*	5.6935**
	−2.2406
*MB*	−0.1491
	(−0.2916)
*YRET*	−0.3125
	(−1.6418)
*LNSIZE*	−3.2304
	(−1.4197)
*AGE*	0.5187
	−1.3272
N	908
F	2.2141

**Indicates a significant correlation at the 0.01 level (two-sided).

## Conclusion

The subjective expression conveyed in the text of financial reports provides valuable incremental information that can somewhat predict the company’s future performance, thereby affecting investors’ views on company prospects and meeting the decision-making needs of investors and business managers. This paper used text-mining technologies such as natural language processing and sentiment analysis to extract and analyze text sentiment from financial reports, and ran a multiple regression analysis to investigate its association with future performance. While investor confidence did not significantly mediate between text net sentiment and future performance, the negative relationship between them was somewhat weakened by earnings per share.

## Data availability statement

The datasets presented in this study can be found in online repositories. The names of the repository/repositories and accession number(s) can be found in the article/supplementary material.

## Ethics statement

The Ethics Committee of Shandong Land Development Group Co., Ltd. reviewed and approved the study.

## Author contributions

NZ contributed to coding and writing the manuscript. JR contributed to data preprocessing. Both authors contributed to the article and approved the submitted version.

## Conflict of interest

NZ was employed by Shandong Tudi Development Group Co., Ltd. JR was employed by company China Construction Bank Shandong Branch.

## Publisher’s note

All claims expressed in this article are solely those of the authors and do not necessarily represent those of their affiliated organizations, or those of the publisher, the editors and the reviewers. Any product that may be evaluated in this article, or claim that may be made by its manufacturer, is not guaranteed or endorsed by the publisher.
